# Histiocytoid cardiomyopathy presenting as sudden death in an 18-month-old infant

**DOI:** 10.1007/s12024-023-00730-2

**Published:** 2023-10-13

**Authors:** Jacob Foster, Sarah Parsons

**Affiliations:** 1 School of Medicine, Cardiff University, Cardiff, Wales UK; 2 Victorian Institute of Forensic Medicine, Melbourne, VIC Australia; 3 Department of Forensic Medicine, Monash University, Melbourne, VIC Australia

**Keywords:** Histiocytoid cardiomyopathy, Sudden death, Infant, Autopsy, Histology

## Abstract

Histiocytoid cardiomyopathy (HC) is an arrhythmogenic disorder, usually involving children under two years of age with a strong Caucasian and female predominance. The disease is fatal in the vast majority and diagnosis is nearly always established at autopsy, but this is only possible with adequate myocardial sampling. Meticulous gross and histological examination of the heart in collaboration with a cardiovascular-trained pathologist maximises the opportunity to make specific diagnoses (and therefore rule out the differentials of SIDS, SUDC and child abuse), guide genetic testing, and inform potentially life-saving medical interventions for blood relations. We present a typical HC case presenting as sudden death, without prodrome, in a previously healthy 18-month-old boy. The disease is characterised histologically by discrete groups of enlarged, polygonal histiocyte-like cells with distinct margins and abundant faintly eosinophilic foamy cytoplasm. Cells often contain coarse granules, microvacuoles and irregular, round nuclei. In our case, dysplastic fascicles were predominantly located immediately deep to the endocardium of the left ventricle. We report our own autopsy findings with histological images, and discuss the expected clinical, morphological and ultrastructural features of the disease.

## Introduction

Histiocytoid cardiomyopathy (HC) is an arrhythmogenic disorder with a distinctive histologic appearance, first described by Voth in 1962 [1]. It usually involves children under two years of age (mean age is 10 to 13 months) and has a strong Caucasian (80%) and female predominance (female:male ratio is 4:1) [2–4]. Although the exact incidence is unknown, HC is presumed to be rare, and fewer than 150 cases have been reported in the literature under varying names. Even then, some cases are probably misdiagnosed as sudden infant death syndrome (SIDS) before 12 months old, or sudden unexpected death in childhood (SUDC) between ages 1 and 18 years; either from inadequate myocardial sampling or parental objection to autopsy.

A global HC registry was established in 1999, and data obtained from this indicates a family tendency in 5% [3]. HC is genetically heterogeneous; and autosomal recessive, maternal and X-linked transmission patterns have been suggested (the latter would explain the apparent female predominance with male prenatal lethality). Whole genome sequencing studies identified nuclear-encoded mitochondrial protein mutations, including distinct *de novo* non-sense mutations in the *NDUFB11* gene on chromosome X [5].

The term “histiocytoid” does not imply histiocytic lineage but rather communicates the cells’ characteristic appearance. Cells are possibly of myocardial precursor or Purkinje origin, but their exact pathogenesis remains unascertained [6]. Because histiocytoid cells have abundant normal or abnormal mitochondria, with genetic alterations resulting in dysfunction of the mitochondrial respiratory chain, many consider HC to be a mitochondrial cardiomyopathy, including the American Heart Association [7, 8].

We present a typical HC case presenting as sudden death, without prodrome, in an 18-month-old infant.

## Clinical history

An 18-month-old Caucasian male was previously in good health and behaving normally on the day of his death. He was fed and put to bed in his cot for a regular afternoon nap. He was located three hours later, unresponsive, with approximately one cup of presumed vomitus on the face, but not in the mouth or airways. Cardiopulmonary resuscitation was attempted but was unsuccessful. The child was declared dead shortly after.

The deceased was born at term to healthy unrelated parents weighing 3.5 kg after an uncomplicated pregnancy. His routine immunisations were up-to-date. There was no significant family history of sudden death, recurrent miscarriages, genetic or cardiac disorders. Families are offered clinical genetic follow-up where sudden cardiac death has occurred in young people, in accordance with European Society of Human Genetics guidelines [9]. However, relatives did not consent to genetic investigation in this case.

## Autopsy

The external examination was unremarkable, and no abnormalities were detected on whole-body computed tomography imaging. The crown-heel length was 870 mm (95^th^ to 97^th^ percentile), and abdominal circumference at the umbilicus was 435 mm [10]. On internal examination, the parietal pericardial surface was smooth, grey and shiny. The cavity contained a small quantity of clear pale-yellow fluid. The heart weighed 69 g (reference range 38–70 g) and the coronary arteries, great vessels, chambers and valves were grossly unremarkable [11]. Tricuspid (54 mm), pulmonary (35 mm), mitral (52 mm) and aortic (34 mm) valve circumferences were within normal limits. Left (11 mm) and right (3 mm) ventricular muscle thickness was also normal. No petechial haemorrhages were identified over the epicardial surface. The myocardium was uniformly red-brown, and the endocardium was thin and translucent, with no grossly discernible diffuse or focal lesions in either.

The pleural cavities contained a small quantity of clear fluid. The lungs appeared congested with scattered petechial haemorrhages over the dorsal and ventral aspects of the lung pleura. The remaining organs were grossly normal.

## Histological examination

**Fig. 1:**
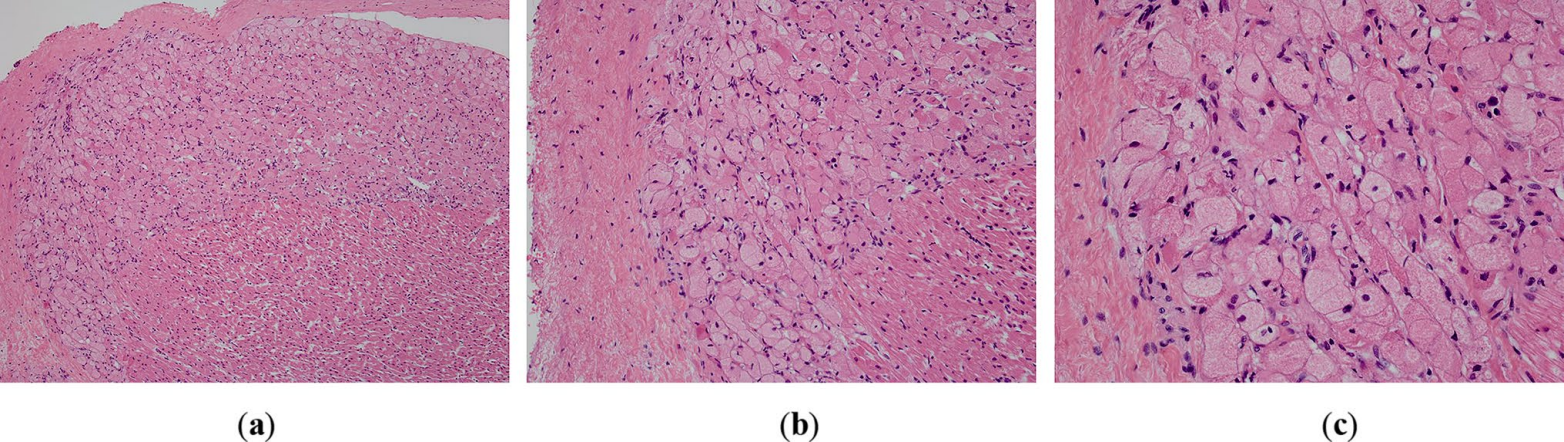
H&E. Left ventricle. 10× (**a**). 20× (**b**). 40× (**c**)

**Fig. 2:**
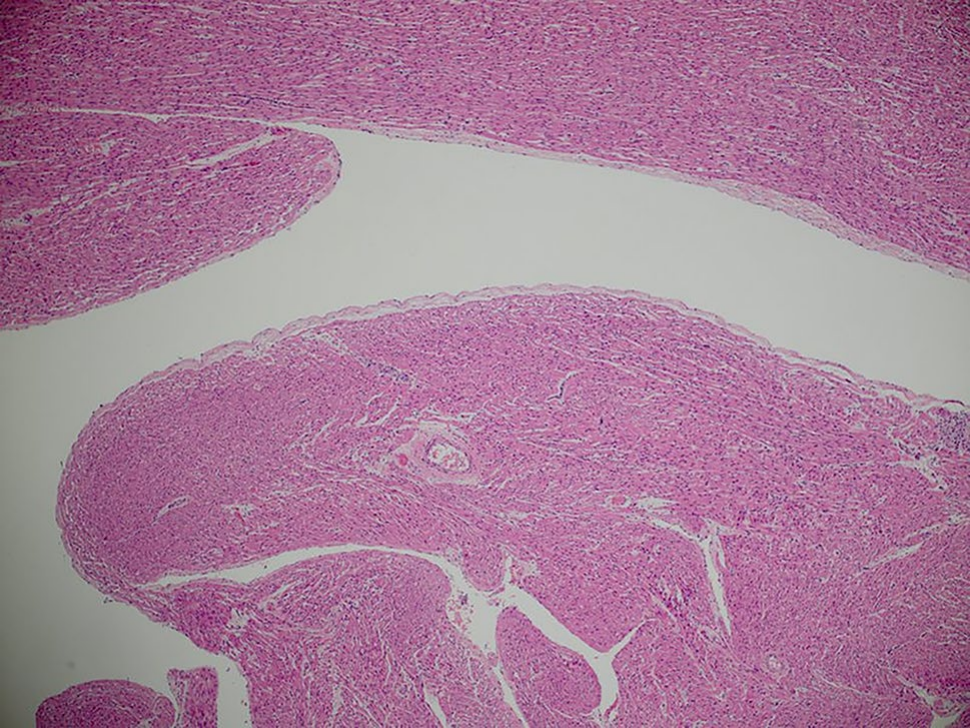
H&E. Right ventricle. 4×

**Fig. 3:**
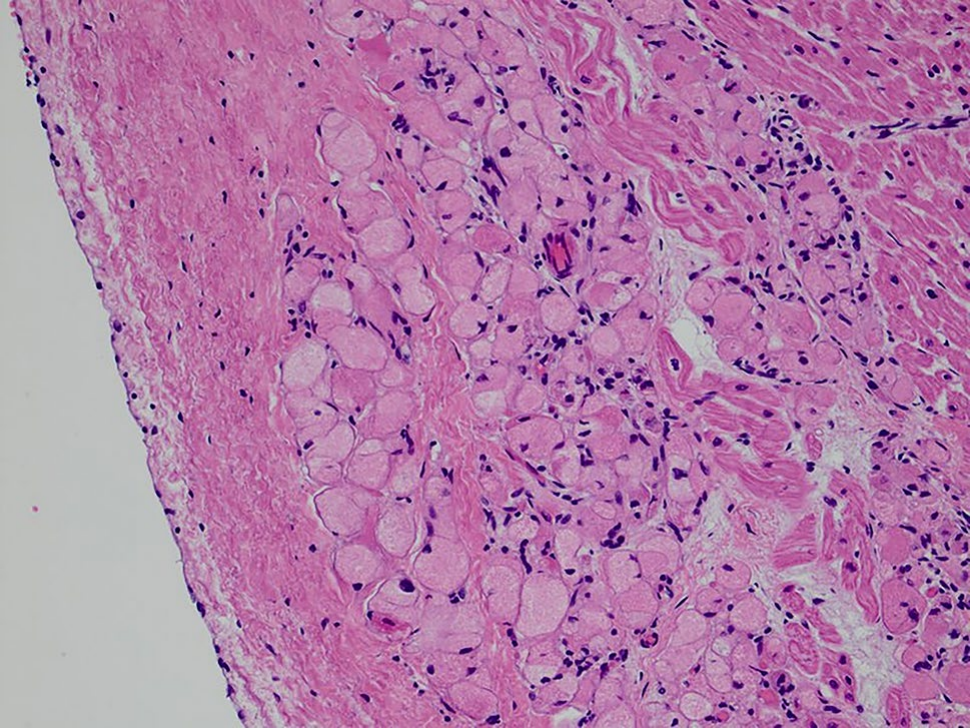
H&E. Interventricular septum. 20×

**Fig. 4:**
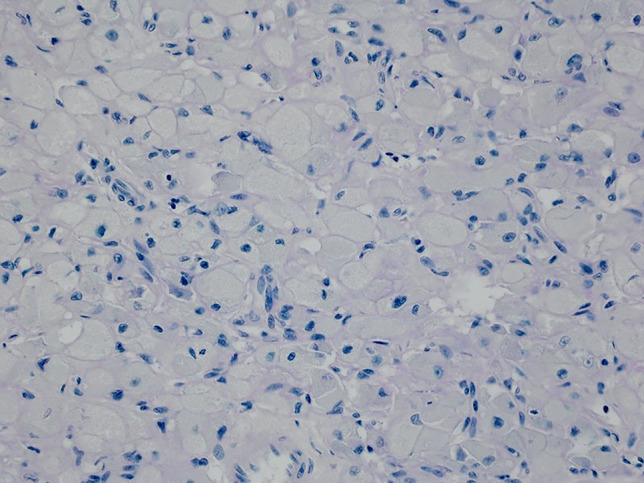
Periodic acid-Schiff stain. Interventricular septum. 40×

**Fig. 5:**
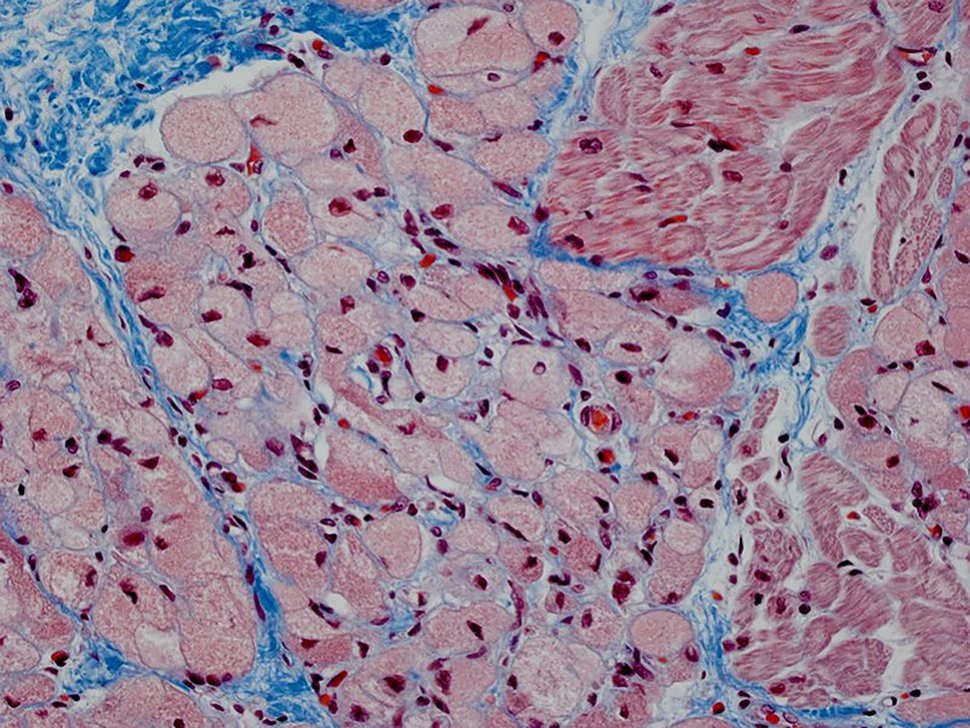
Masson’s trichrome stain. Interventricular septum. 40×

Formalin-fixed, paraffin-embedded, haematoxylin and eosin-stained sections from the heart showed multiple discrete groups of enlarged polygonal histiocyte-like cells with distinct margins and abundant faintly eosinophilic foamy cytoplasm, containing coarse granules and irregular, round nuclei. Some of these cells contained numerous microvacuoles. There were no “spider cells” suggestive of other hamartomatous processes, such as rhabdomyomas. Dysplastic foci were predominantly located immediately deep to the endocardium of the left ventricle (Fig. 1), but also within the myocardium of the septum at the base of the heart, just inferior to the atrioventricular node. Clusters of histiocytoid cells were also present immediately deep to the right ventricular endocardium but were fewer (Fig. 2). Other clusters were seen towards the base of the heart, including at the most superior edge of the muscular interventricular septum (Fig. 3). The remaining myocardium was normal, with no myocyte degeneration or significant inflammation. Periodic acid-Schiff stains were negative with and without diastase pre-digestion (Fig. 4). Masson’s trichrome demonstrated focal endocardial fibroelastosis overlying the lesions (Fig. 5).

All other organs were within normal histologic limits, and histiocytoid cells were not observed in any extracardiac site.

## Discussion

HC follows a fulminant clinical course, usually with dilated cardiomyopathy (in 95%), a primary dysrhythmia (in 70%) or sudden death (in 20%) [12]. Some cases also report a short flu-like prodrome before death. The disease is fatal in the vast majority, and diagnosis is nearly always established at autopsy.

Macroscopically, HC is characterised by yellowish nodules with diameter 1 to 15 mm present in the myocardium (in 75%), endocardium or, rarely, the epicardium [4, 13]. In some, like the present case, pathologic tissue is not macroscopically obvious. Cardiomegaly (in 95%) has been described previously, but heart weight can be within normal limits, as in this case [2]. Associations with additional cardiac anomalies (most notably, left ventricular hypertrabeculation, non-contraction and endocardial fibroelastosis) and extracardiac anomalies have also been reported in 16% and 17% of cases, respectively [2, 14].

The microscopic appearance from our case is typical of HC and results from a pathologic process involving fibril loss and marked mitochondrial hyperplasia. The outcome is rounded, enlarged cells of myocytic origin with granular eosinophilic cytoplasm, resembling histiocytes. Cells are arranged in fascicles, giving a pseudonodular appearance, with a distribution mimicking that of the cardiac conduction system. Ultrastructurally, the abnormal cells contain scant myofibrils, which appear distorted and fragmented [15]. The cells also resemble cardiac conduction tissue: they lack a T-tubule system, harbour large numbers of mitochondria, and interact via desmosomes.

## Conclusion

In contrast to other published HC reports, we presume death in our case may have occurred relatively early in the disease process, given the patient’s young age and absence of grossly apparent nodules or cardiomegaly at autopsy.

Around 19% of sudden natural deaths in young people aged 1 to 13 years are cardiac in origin [16]. Due to their age, unexplained circumstances and lack of medical history, these cases are typically referred to the coroner (or equivalent) for medicolegal investigation. They therefore represent a significant proportion of forensic pathologists’ workload. Meticulous gross and histological examination of the heart in collaboration with a cardiovascular-trained pathologist maximises the opportunity to make specific diagnoses (and therefore rule out the differentials of SIDS, SUDC and child abuse), guide genetic testing, and inform potentially life-saving medical interventions for blood relations [17].
